# Agroforestry Practices Promote Biodiversity and Natural Resource Diversity in Atlantic Nicaragua

**DOI:** 10.1371/journal.pone.0162529

**Published:** 2016-09-08

**Authors:** Seeta A. Sistla, Adam B. Roddy, Nicholas E. Williams, Daniel B. Kramer, Kara Stevens, Steven D. Allison

**Affiliations:** 1 School of Natural Science, Hampshire College, Amherst, Massachusetts, 01002, United States of America; 2 Department of Ecology and Evolutionary Biology, University of California Irvine, Irvine, California, 92697, United States of America; 3 School of Forestry & Environmental Studies, Yale University, New Haven, Connecticut, 06511, United States of America; 4 Department of Anthropology, University of California Santa Barbara, Santa Barbara, California, 93106, United States of America; 5 Department of Environmental Studies, University of Colorado Boulder, Boulder, Colorado, 80309, United States of America; 6 Department of Fisheries and Wildlife, Michigan State University, East Lansing, Michigan, 48824, United States of America; 7 Department of Earth System Science, University of California Irvine, Irvine, California, 92697, United States of America; Chinese Academy of Forestry, CHINA

## Abstract

Tropical forest conversion to pasture, which drives greenhouse gas emissions, soil degradation, and biodiversity loss, remains a pressing socio-ecological challenge. This problem has spurred increased interest in the potential of small-scale agroforestry systems to couple sustainable agriculture with biodiversity conservation, particularly in rapidly developing areas of the tropics. In addition to providing natural resources (i.e. food, medicine, lumber), agroforestry systems have the potential to maintain higher levels of biodiversity and greater biomass than lower diversity crop or pasture systems. Greater plant diversity may also enhance soil quality, further supporting agricultural productivity in nutrient-limited tropical systems. Yet, the nature of these relationships remains equivocal. To better understand how different land use strategies impact ecosystem services, we characterized the relationships between plant diversity (including species richness, phylogenetic diversity, and natural resource diversity), and soil quality within pasture, agroforests, and secondary forests, three common land use types maintained by small-scale farmers in the Pearl Lagoon Basin, Nicaragua. The area is undergoing accelerated globalization following the 2007 completion of the region’s first major road; a change which is expected to increase forest conversion for agriculture. However, farmer agrobiodiversity maintenance in the Basin was previously found to be positively correlated with affiliation to local agricultural NGOs through the maintenance of agroforestry systems, despite these farmers residing in the communities closest to the new road, highlighting the potential for maintaining diverse agroforestry agricultural strategies despite heightened globalization pressures. We found that agroforestry sites tended to have higher surface soil %C, %N, and pH relative to neighboring to secondary forest, while maintaining comparable plant diversity. In contrast, pasture reduced species richness, phylogenetic diversity, and natural resource diversity. No significant relationships were found between plant diversity and the soil properties assessed; however higher species richness and phylodiversity was positively correlated with natural resource diversity. These finding suggest that small, diversified agroforestry systems may be a viable strategy for promoting both social and ecological functions in eastern Nicaragua and other rapidly developing areas of the tropics.

## Introduction

Together, crop- and pasture-lands comprise one of the largest biomes on earth, representing ~ 40% of global terrestrial area [1]. While agricultural innovations have greatly increased food production, they have also caused extensive environmental degradation; nearly half of global croplands are impacted by soil erosion, declines in soil fertility, reduced biodiversity, and other socio-ecological concerns [2–5]. In the tropics, deforestation for agricultural expansion accounts for 8% of anthropogenic carbon dioxide (CO_2_) emissions–nearly all global land-use change emissions–and is the primary cause of species extinctions worldwide [6–8]. Coupling sustainable agriculture to biodiversity conservation through small, diversified farms, such as those typified by traditional tropical agroforestry ecosystems, may be a viable complementary land use strategy in rapidly developing areas of the tropics [1,3,4,9–12]. Despite the potential socio-ecological benefits of agroforestry systems and the possibility for them to support conservation efforts in ecologically fragile areas of high biodiversity, tropical conservation policy remains dominated by efforts to reduce intact forest conversion and promote natural reforestation in lieu of supporting more socio-ecologically integrative practices [3,13–15]. Increasing our understanding of the ecosystem service benefits of traditional agroecosystems could help encourage policy that supports a broader range of conservation objectives [16].

Agroforestry ecosystems that incorporate perennial trees into agriculture, such as those typified by smallholder farmer-dominated areas in the tropics (typically 0.01 to 5 ha), can be a fundamental component of both biodiversity conservation and socio-ecological resilience [10,12,17–20]. In addition to provisioning natural resources (i.e. food, medicine, and building materials), agroforestry ecosystems have the potential to maintain higher levels of biodiversity and greater biomass than conventional agriculture. Relative to monoculture or pasture techniques, agroforestry ecosystems may also enhance soil quality, including C content and nutrient status, by increasing litter inputs and soil organic matter accumulation [2,3,17,21–23]. In many tropical systems, agricultural productivity is constrained by low nutrient availability due to highly leached, acidic soils; this problem is amplified because many subsistence farmers in these areas often cannot access mineral fertilizers [24].

Positive relationships between plant diversity and ecosystem functions such as C sequestration—which can be driven by either niche complementarity or the greater likelihood of including functionally-important species in more diverse assemblages—have been identified in a number of model ecosystems [25–27], although these relationships are complex and positive species/functional diversity relationships are not always observed [21,28]. Both plant community functional diversity and phylogenetic diversity, rather than simply the number of taxonomic units (e.g. species or functional group richness), appear to underlie observed biodiversity–ecosystem-service relationships [29,30]. However, the nature of these relationships remains equivocal in human-managed systems, such as smallholder agroecosystems typical in developing areas of the tropics [9,21,31–33].

**Fig 1:**
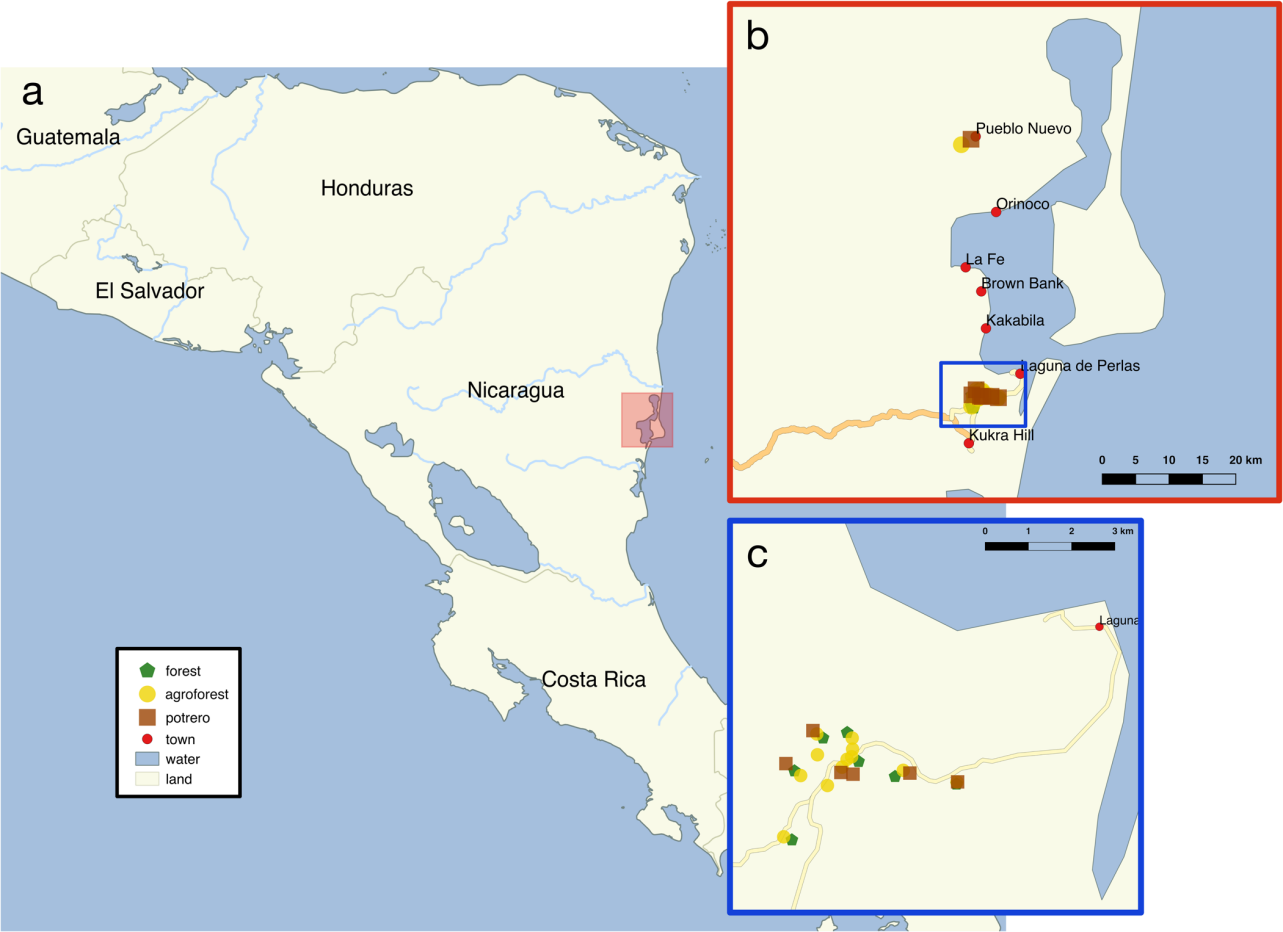
Maps of the study region. Nicaragua (a) with an inset (b) defining the Pearl Lagoon Basin. Sampled plots (c) are shown by points colored by land use type: secondary forest (green), agroforest (yellow), pasture (red).

We characterized the relationships between common smallholder land use strategies, plant diversity, and soil quality in the Pearl Lagoon Basin, Nicaragua (Fig 1) to better understand how these strategies impact a variety of ecosystem functions and properties. The Basin, which is situated within the Mesoamerica Biodiversity Hotspot, is an area historically characterized by a matrix of agroforestry systems and unmanaged secondary forest [34] and is on the eastern edge of a front of rapid pasture expansion [35]. The area is also undergoing accelerated globalization following the 2007 completion of the region’s first major road, which connects the Basin with the rest of Nicaragua [36].These changes are expected to increase forest conversion for agriculture, particularly pasture [34]. Intriguingly, a recent study in the Basin found that farmer agrobiodiversity maintenance is positively correlated with affiliation to local agricultural NGOs through the maintenance of agroforestry systems, despite these farmers residing in the communities closest to the new road [37]. These findings suggest that despite heightened globalization pressures, the potential for supporting more ecologically diverse agricultural strategies can be maintained with culturally-specific land use norms [38].

**Fig 2:**
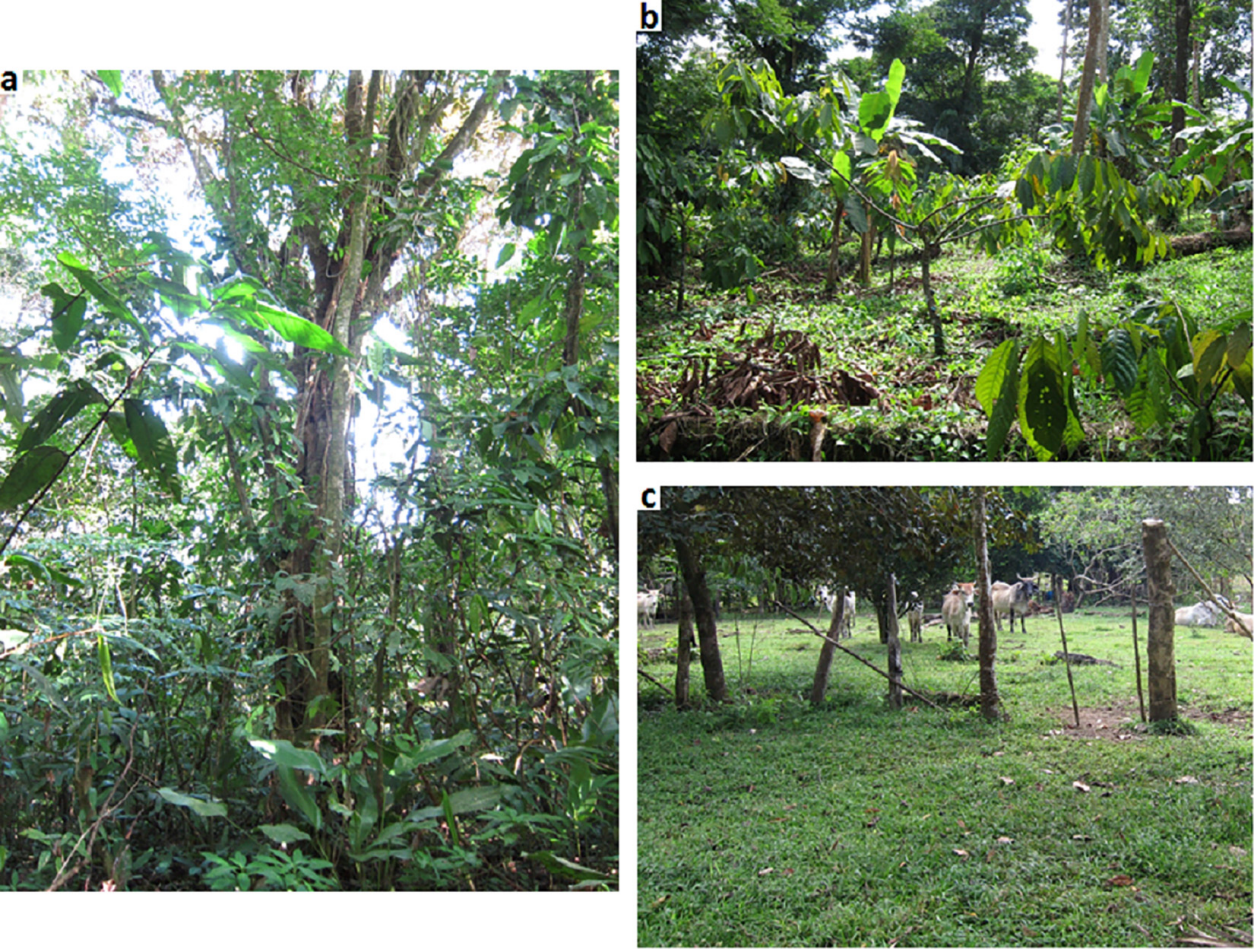
Images of landscapes sampled. Secondary forest (a), agroforest (b), pasture (c) in the Pearl Lagoon Basin, Nicaragua.

Limited understanding of the interrelated effects of land use strategy (agroforestry vs. pasture vs. unmanaged secondary forest) on soil quality and plant diversity in this system hinders our ability to project how accelerated expansion of agroforestry versus pastureland will impact the region’s terrestrial ecosystem services. Further, characterizing how land use affects natural resource availability is fundamental to assessing whether traditional agroforestry practices are comparable to natural reforestation within this system. To help fill these knowledge gaps, we characterized how three land-use types common in Atlantic Nicargua (agroforestry, pasture, secondary forest, Fig 2) affect total (wild and cultivated) plant species diversity and a suite of soil properties. We also estimated potential natural resource diversity provided by plants in each land-use type within four categories (food, medicine, lumber, and other uses, collectively termed ‘natural resources’). We hypothesized that: 1) secondary forests would be the most species-rich and phylogenetically-diverse system, while pasture would support the lowest diversity; 2) soil nutrient status would positively correlate with species-richness and phylogenetic diversity; and 3) agroforestry ecosystems would support the highest functional diversity of ecosystem services, while pasture would have the lowest functional diversity. Identifying how land use affects biodiversity and ecosystem services from both a social and ecological perspective is fundamental to assessing whether traditional agroforestry practices might serve as a tractable alternative to unmanaged reforestation in Atlantic Nicaragua and similar systems.

## Methods

### Site description

The Territory of the 12 Indigenous & Afro-descendant Communities of the Pearl Lagoon Basin, Región Autónoma de la Costa Caribe Sur (RACCS) encompasses 5200 km^2^ in Atlantic Nicaragua. The Basin is located within the Mesoamerican biodiversity hotspot, which includes all subtropical and tropical ecosystems from central Mexico to the Panama Canal [36]. Our focal study area was characterized by Central American Atlantic moist forest, which is the dominant forest ecoregion along the Nicaraguan Atlantic Coast. The Pearl Lagoon region moist forest is characterized by a history of disturbances including the Class IV hurricane Joan (1989) and a patchwork of previous agroforestry sites [36]. Atlantic Nicaragua has a tropical wet climate and is characterized by a wet season with an average rainfall (MAP) exceeding 500 cm with distinct wet (May—November) and dry (December—April) seasons and an average temperature (MAT) of 26°C [39]. The region is dominated by highly weathered, iron- and aluminum-oxide rich ultisols [39].

The Pearl Lagoon Basin is predominantly inhabited by indigenous and afro-descendant populations who utilize marine and aquatic fishery resources in conjunctions with agricultural and forest resources to meet their subsistence needs. Basin indigenous and afro-descendant farmers commonly maintain a wide variety of ethnobotanically-important cultivated and wild species for food, medicinal, timber, fuel, ornamental, and other uses [37]. Agroforestry systems in the Pearl Lagoon Basin are typified by small-scale subsistence farms characterized by a diversity of annual crops interspersed with perennial fruit trees. Common cultivated plants include: citrus varietals, coconuts (*Cocos nucifera*), supa (*Bactris gasipaes*), cacao (*Theobroma cacao*), breadfruit (*Artocarpus altilis*), mango (*Mangifera indica*), avocado (*Persea americana*), varieties of plantain and banana (*Musa sp*.); pineapples (*Ananas comosus*), and starchy roots, including cassava (*Manihot esculenta*) and dasheen (*Colocasia esculenta*). In addition to agroforestry systems, farmers in the region also commonly maintain homegardens for daily use as well as small cattle pastures for personal consumption or local market. Farm land is interspersed with secondary forest held in communal tenure [37].

This type of agroforestry system has been used by Basin afro-descendant and indigenous residents for at least a century [37,40,41]. Mestizo farmers who are part of a wave migration from Nicaragua’s highland regions rely on market-oriented cattle farming leading to the conversion of forest to pastureland and accelerating forest fragmentation. Following the end of the Nicaraguan civil war (1979–1987), cultural diffusion has increased the prevalence of cattle ranching among afro-indigenous farmers [36,37]. However, local agricultural NGOs and Nicaraguan governmental organizations are working to mitigate land conversion by promoting local agroforestry practices through interaction with individual farmers, which are correlated with greater agrobiodiversity maintenance at the farm-level [37].

### Farm identification

We identified thirteen farms in the Pearl Lagoon Basin that included agroforest (n = 17 sites), adjacent secondary forest (n = 8 sites), and pasture sites (n = 7 sites). Sample farms were identified by conducting interviews with farmers to determine their land use practices (method approved by UCSB Human Subjects Committee, submission ID: 13–0324). Literacy rates are low in the region, as such, verbal consent to sample each farmer’s land was obtained from participants. Farmer consent was digitally recorded by the researchers (S. Sistla, N. Williams). Farmers were asked if they had cleared forest on their site, when their sites were established, and if their pasture was actively grazed. Areas within any of the three land use types that had been burned within the past decade, were subject to external fertilizer and/or pesticide application, or had been planted less than a decade ago were excluded from the survey. This time period was chosen because previous studies in the region found that a 5-year interval following disturbance from hurricane or agricultural abandonment was sufficient to allow pioneer species to establish [42,43].

All sample farms included at least one agroforestry site, and no farm had more than one pasture area. Specific agroforestry land use strategies (coconut-dominated, cacao-dominated, plantain-dominated, mixed fruit) were identified through the farmer survey and sampled as separate agroforestry sites within each farm. All agroforestry sites in a given farm that met the sampling criteria were included in the plant and soil surveys.

### Plant surveys

Plant surveys and soil sampling were conducted during the dry season from February through April, 2013. At each agroforestry, pasture, and secondary forest area, four haphazardly selected 3x3 m plots, whose location was recorded with GPS, were sampled within each land use type. Understory species were sampled from 1-m^2^ quadrats centered within the 3x3 m plots [44,45]. Within each 1-m^2^ quadrat, all identifiable plant species (including sub-adults) were recorded by common name in Spanish or Creole with assistance from a community member identified through a local agricultural NGO as an expert on local plants. Digital photographs were taken of each quadrat, and unknown plant species were individually photographed for post-sampling identification to the family level; unidentifiable species were recorded as unknown morphotypes. Species abundance was not considered in these analyses due to the dense growth patterns of many observed species, particularly grasses and herbaceous species.

### Natural resource diversity

Potential human uses for known species (i.e. natural resource diversity) were identified through ethnobotanical literature focused on eastern Nicaragua. Plant uses were cross-validated between the ethnobotanical literature and ethnobotanical surveys conducted in tandem with our study [37]. Natural resource uses were defined as: “timber”, “medicinal”, “food”, and “other” (which included dyes and tannins, fuel, animal forage, ornamental, and household utensils) [40,46–51]. Each identified species was categorized as either serving or not serving each of those 4 categories; a species’ ‘total functionality’ under these criteria could therefore range from 0 (no use) to 4 (all uses).

Potential natural resource diversity for each quadrat was estimated in three ways. First, the total number of functions was summed across species in a quadrat, which we termed *F*_*T*_. For example, a community with two species that both have two functions would have a *F*_*T*_ equal to four, regardless of whether the two functions were the same or different. The *F*_*T*_ metric allows for functionally redundant communities that may nonetheless be functionally limited (i.e. many species serving the same few functions) to be equivalent to communities that have low redundancy but high functional diversity (i.e. many species that do not have common functions). Second, we divided *F*_*T*_ by the number of species in each quadrat to calculate the average functional value of a species in that quadrat, termed here $\bar{F}$. This accounts for differences in species richness that can inflate F_T_ such that quadrats with a high *F*_*T*_ would be composed of species that each serve many functions. Third, we calculated an analog of Simpson’s Index of Diversity for potential natural resource diversity as:

$$D=1-\sum {\left(\frac{F_{i}}{F_{T}}\right)}^{2}$$

where *F*_*i*_ is the total number of functions of a particular species and *F*_*T*_ is the total number of all functions of all species, as defined above. Similar to other applications of Simpson’s Index of Diversity, *D* is scaled between 0 and 1, with a larger *D* value equivalent to high functional diversity. This functional analog to the traditional Simpson’s Index of Diversity metric measures the probability that any two species selected from a community will have the same function. These three metrics, therefore, have different and complementary properties that allow for discrimination between functional diversity, functionally redundancy, and the average functional value of different communities. We chose to focus on species richness rather than abundance to avoid confounding non-productive (i.e. non-fruit bearing or immature lumber species) with productive individuals of a given species for a given resource.

### Plant phylodiversity

A regional species pool was assembled from (ethno)botanical assessments of the region, sociological surveys of plants growing on farms, and supplemented by our plant surveys. Plants identified in our surveys but not present in either the botanical or sociological assessments were identified to the family level and scored as distinct morphotypes. From this combined list, we generated a family-level phylogeny using the megatree in Phylomatic [52] and manually added basal land plants, based on current phylogenetic understanding, that were not in the Phylomatic megatree but that appeared in our plant surveys.

This master tree, which was used for all subsequent phylogenetic analyses, constituted 468 total species, of which 136 occurred in the plant surveys in the present study. We did not scale branch lengths proportional to time and instead calculated branch lengths proportional to diversity [53]. Incorporating more accurate branch length estimates does not necessarily improve estimates of phylodiversity [54]. We calculated three metrics of phylodiversity using the R package *picante* (v. 1.6–2) [55]: Faith’s Index (hereafter ‘phylodiversity’), the Net Relatedness Index (NRI), and the Nearest Taxon Index (NTI). Faith’s Index describes the phylogenetic diversity of each community based on the total phylogenetic branch lengths in each community and is correlated with species richness while NRI characterizes the average phylogenetic distance between species in a community and NTI characterizes the average phylogenetic distance to the most closely related species in the community. NRI and NTI showed similar patterns and so, for brevity, we focus only on NRI. Because our master tree included many more species than were observed in the plant surveys, estimates of NRI are deflated and will resemble more phylogenetic overdispersion (less clustering) than if we had sampled from a phylogeny composed solely of the 136 species observed in the plant surveys. Although this biases our estimates of NRI towards overdispersion (as compared to other studies), the absolute differences among our plots are still comparable to each other.

To account for species turnover among land use types, we used the aggregated species lists for each land use type to further explore how management strategy impacted biodiversity and species dispersal. We contrasted plant species composition among land-use types using a Jaccard similarity coefficient, which compares species presence/absence data for two sample sets (e.g. land-use types). The Jaccard similarity coefficient is calculated as:

$$J=\frac{A\cap B}{A\cup B}$$

where A and B are the species sets in each of two land use types.

### Soil sampling and analyses

Two 10-cm deep, 5-cm diameter soil cores within the 1-m^2^ quadrat were collected and pooled for all subsequent analyses. Wet weight of the total plot-level pooled duplicate cores and a sub-sample from the pooled cores was recorded the day of sampling. Subsamples were then air dried and sealed. Due to the remote conditions of the study site, subsequent analyses were carried out at the Iowa State University Soil and Plant Analysis Laboratory. The soil subsamples were dried to a constant mass, and total organic carbon (C) and total nitrogen (N) concentrations were determined by combustion using a C/N analyzer. Available soil phosphorus (P) was estimated using the Bray and Kurtz P-1 test [56]. Soil pH was measured using a 1:1 soil/slurry. Bulk density was determined by calculating the total dry weight of the cores and dividing by the total core volume.

### Statistical analyses

The impact of land use type on diversity was analyzed using nested mixed models in R (*lmer* package) [57], with land use type (secondary forest, agroforest, pasture) coded as a fixed factor and specific land use strategy (agroforestry type) nested within farmer, which was coded as a random effect. No significant difference in species richness (p = 0.7), phylodiversity (p = 0.7), or NRI (p = 0.9) were identified between specific agroforestry land use strategies, so these strategies were pooled under the land use type ‘agroforest’ for all subsequent analyses to simplify interpretation and the potential for application. Post hoc comparisons of means were performed with Tukey's honest significant difference (HSD) test. The relationships between diversity metrics (species richness and phylodiversity) and soil qualities (%C, %N, P, pH, and bulk density) and plant natural resource diversity were tested using a linear mixed effects model (LME).

## Results

### The influence of land use on plant species diversity

**Fig 3:**
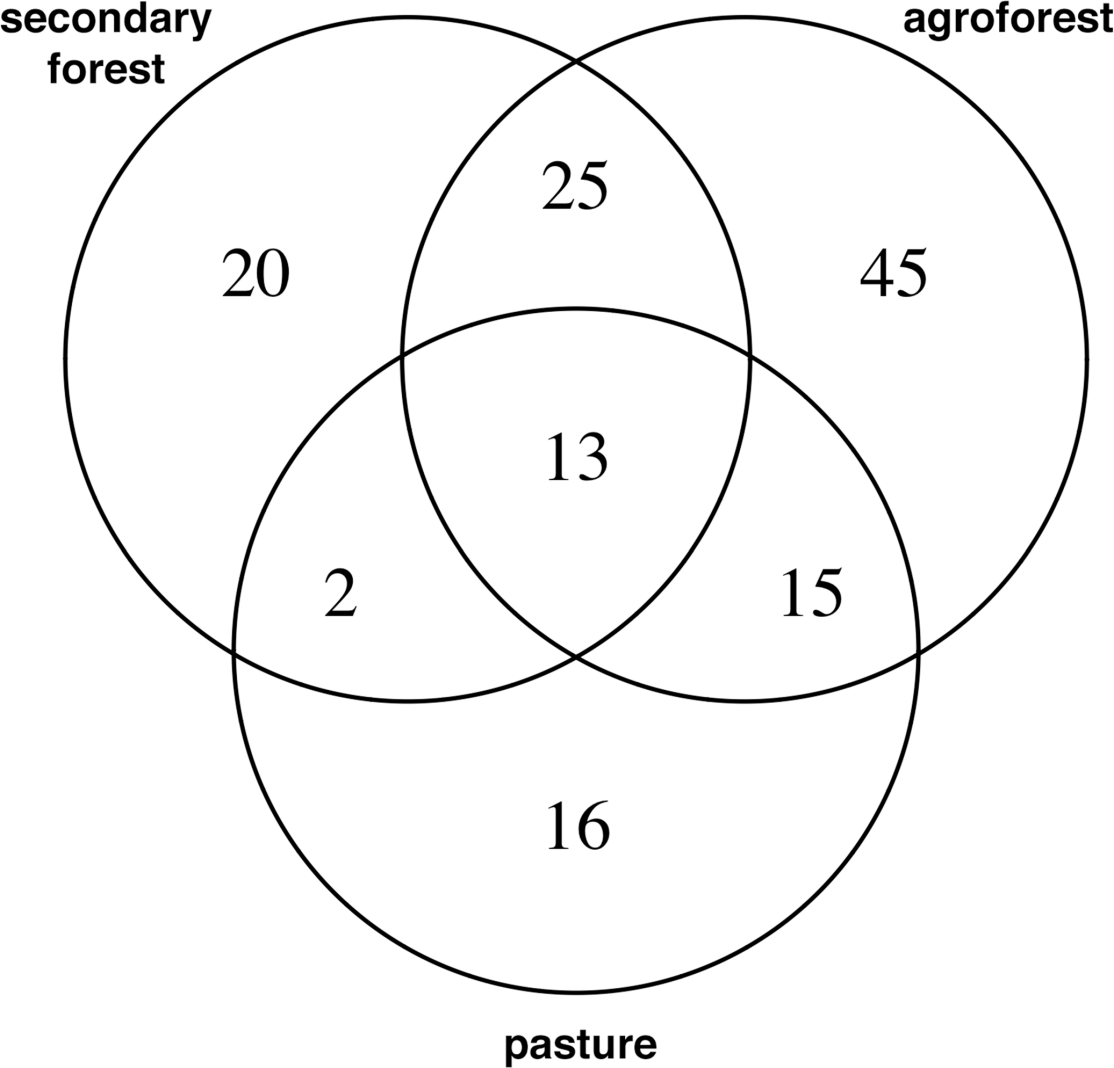
A Venn diagram of the number of morphospecies overlap between the different land use types.

Across land use types, we identified a total of 136 species to the family level or below. Between 0 and 4 unidentifiable morphospecies were found across sample quadrats with no difference in average unknown species among land use types; unidentified species were not included in the phylodiversity metrics. The total number of morphospecies per quadrat (m^-2^) ranged from 17 morphospecies (in the secondary forest) to 1 (in the pasture). We note that pasture systems tended to be both species-poor and extremely small statured (typically < 5 cm height). Secondary forests and agroforests shared the greatest number of species (38 species with Jaccard similarity (*J*) = 0.32), while secondary forest and pasture shared 11% of their species (15 species with *J* = 0.17). Agroforests and pasture shared 20.5% of their species (28 species with *J* = 0.24; Fig 3).

**Fig 4:**
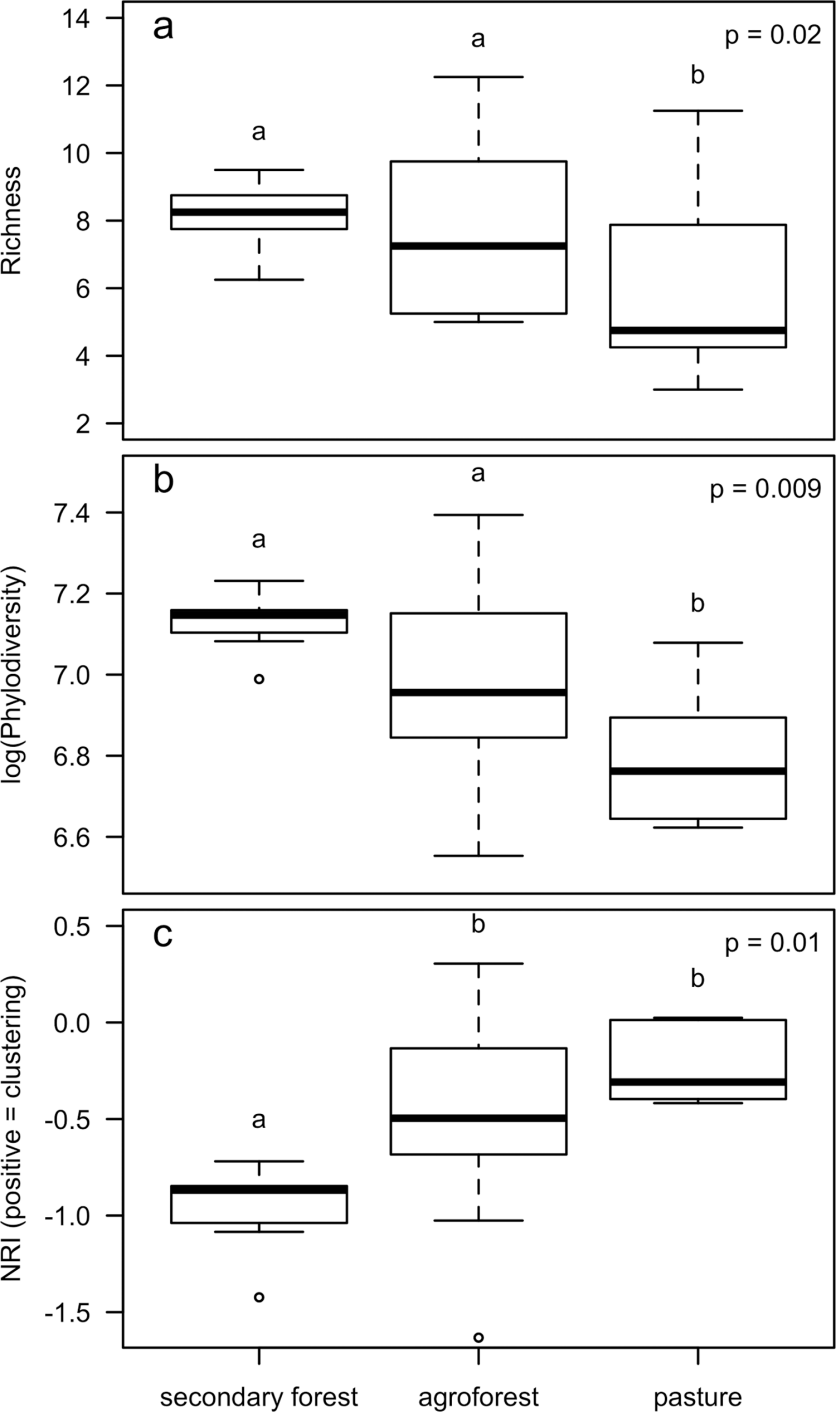
Land use effects on plant species diversity. Effect of land use type (secondary forest (N = 8), agroforest (N = 17) and pasture (N = 7)) on three metrics of diversity: species richness (a), phylodiversity (b), and Net Relatedness Index (C). Non-overlapping letters signify significant difference at α ≤ 0.05.

Land use type significantly affected plant diversity across all metrics tested (Fig 4), including species richness (p = 0.02; this effect was equivalent when unidentified species were included), Faith’s phylodiversity (p = 0.009), and net relatedness index (NRI, p = 0.01). Secondary forest and agroforest species richness per m^2^ was 8.14 ± 0.15 and 7.97 ± 0.15, respectively, while pasture species richness was 6.18 ± 0.6. Tukey HSD post hoc comparisons indicated that secondary forest and agroforest were significantly more diverse in terms of species richness and Faith’s phylodiversity (1248.7 ± 13.2, 1095.1 ± 12.4, respectively) than pasture (911.6 ± 25.7), but did not significantly differ from each other. In contrast, secondary forest had a significantly lower NRI (-0.97 ± 0.03) than either agroforest or pasture (-0.50 ± 0.03, -0.22 ± 0.03, respectively), which did not significantly differ from each other.

### The influence of land use on plant-based natural resource diversity

**Fig 5:**
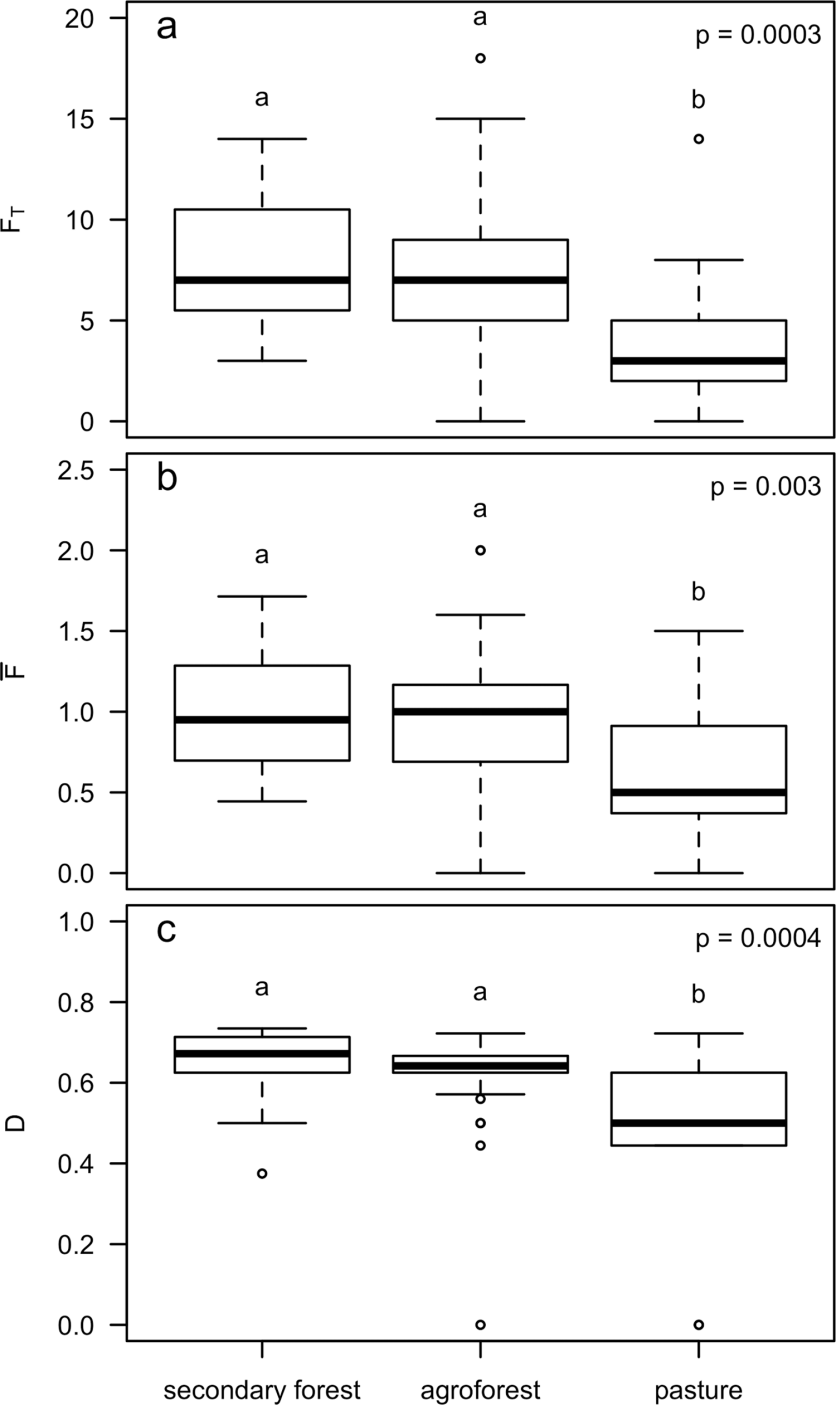
Land use effects on natural resource diversity. Effect of land use type (secondary forest (N = 8), agroforest (N = 17) and pasture (N = 7)) on total natural resource diversity (*F*_*T*_) (a), average natural resource diversity $(\bar{F)}$ (b), and Simpson’s Index of Natural Resource Diversity (*D*) (c). Non-overlapping letters signify significant difference at α ≤ 0.05.

Land use type also significantly affected total potential natural resource diversity (*F*_*T*_; p = 0.0003), average functional value of each species ($\bar{F}$; p = 0.003), and Simpson’s Index of Functional Diversity (*D*; p = 0.0004; Fig 5). Tukey HSD post hoc comparisons indicated that secondary forest and agroforest had a significantly greater *F*_*T*_, $\bar{F}$, and *D* than pasture, but did not significantly differ from each other.

**Fig 6:**
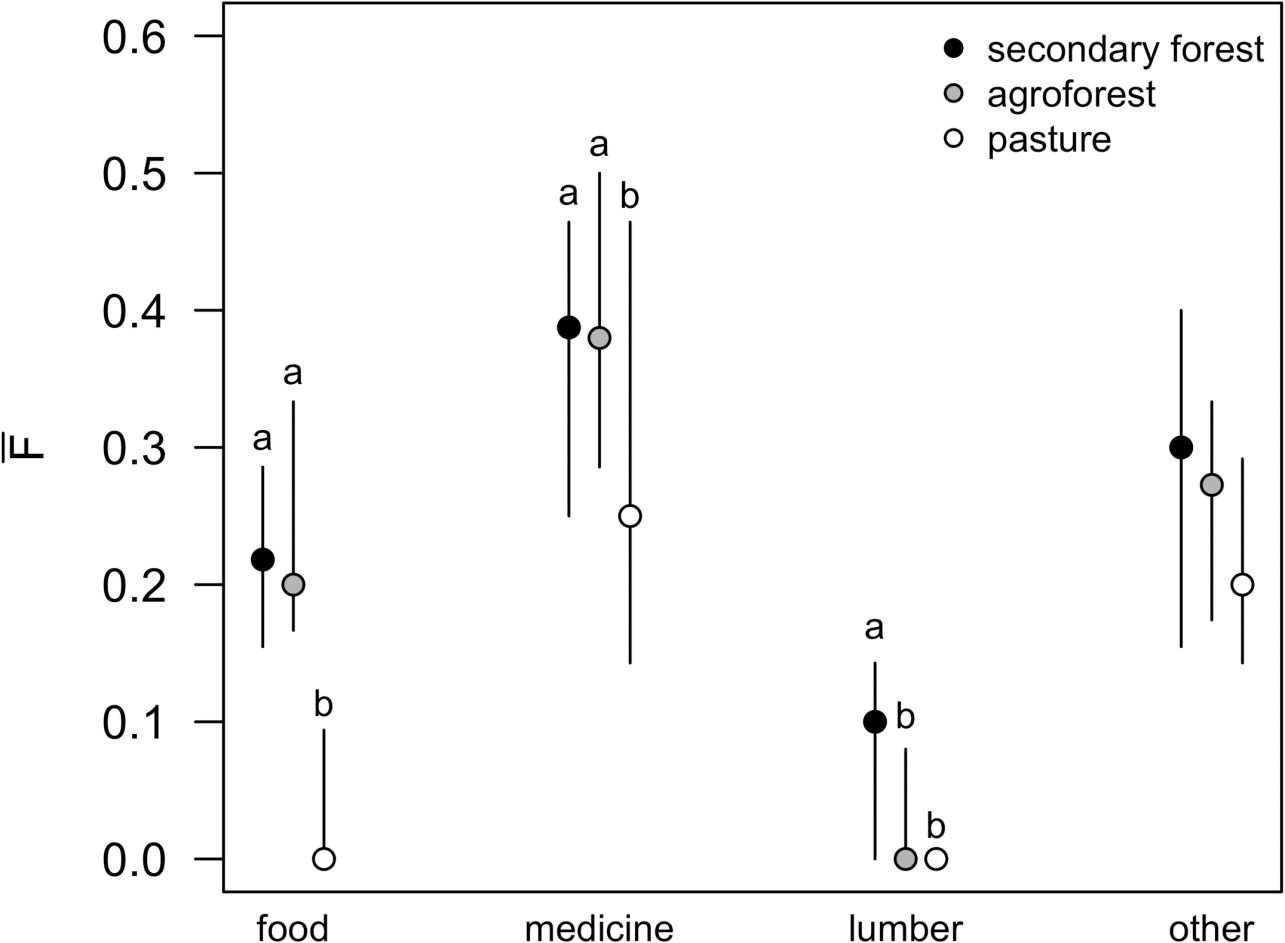
The proportion of plant species used for various social functions in each land use type. The proportion of species in each land use type (secondary forest (N = 8), agroforest (N = 17) and pasture (N = 7)) that are used for: food, medicine, lumber, and other. Non-overlapping letters signify significant difference at α ≤ 0.05 for each functional group.

**Fig 7:**
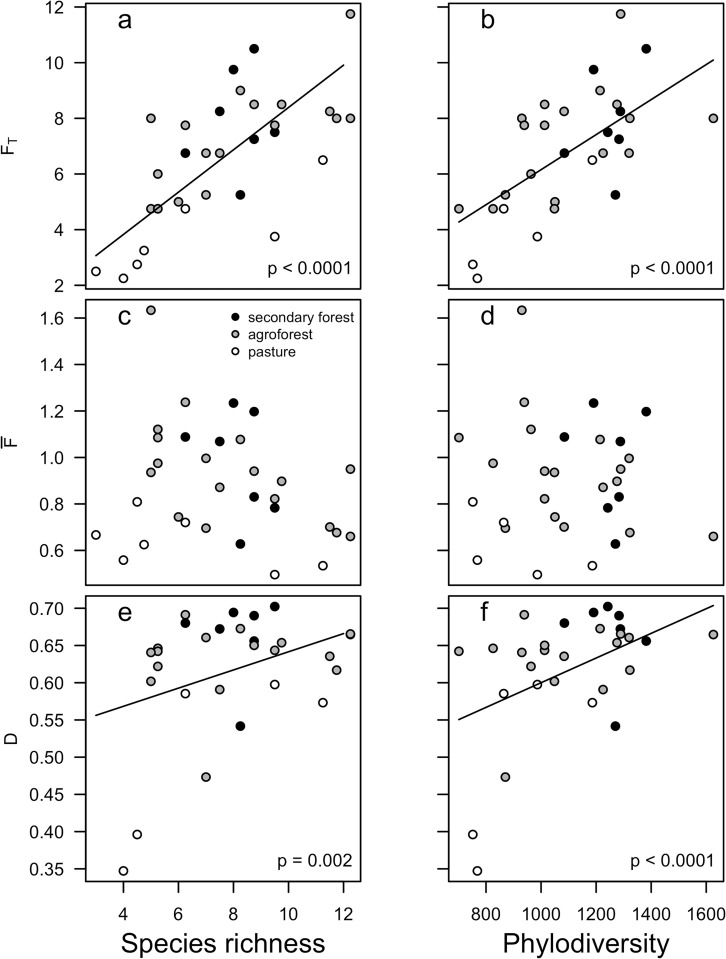
Relationships between plant diversity and natural resource diversity. Effect of species richness and phylodiversity on *F*_*T*_ (a,b), $\bar{F}$ (c,d), and *D* (e,f). Non-overlapping letters signify significant difference at α ≤ 0.05.

Paralleling this pattern, for three of the four specific functions assessed, land uses differed significantly in the average potential functional value per species: food (p < 0.0001), medicine (p = 0.03), and lumber (p = 0.006; Fig 6). Tukey HSD post hoc comparisons indicated that secondary forest and agroforest had higher average food and medicinal value than pastureland, but did not significantly differ from each other in either function. Secondary forest was more functionally valuable for lumber than both pasture and agroforest, which did not differ from each other. The proportion of species in a given community that had one or more potential natural resource functions did not vary for agroforest, whereas secondary forest and pasture were predominantly composed of one- or two-function species with almost no three-function species (S1 Fig). Across land use types, more biodiverse communities were also more functionally diverse (Fig 7). Species richness and phylodiversity had significant positive effects on *F*_*T*_ (both p < 0.0001; Fig 7A and 7B) and on *D* (p = 0.002 and p < 0.0001, respectively; Fig 7E and 7F), but no effect on $\bar{F}$ (Fig 7C and 7D).

### Relationships between land use and soil qualities

**Fig 8:**
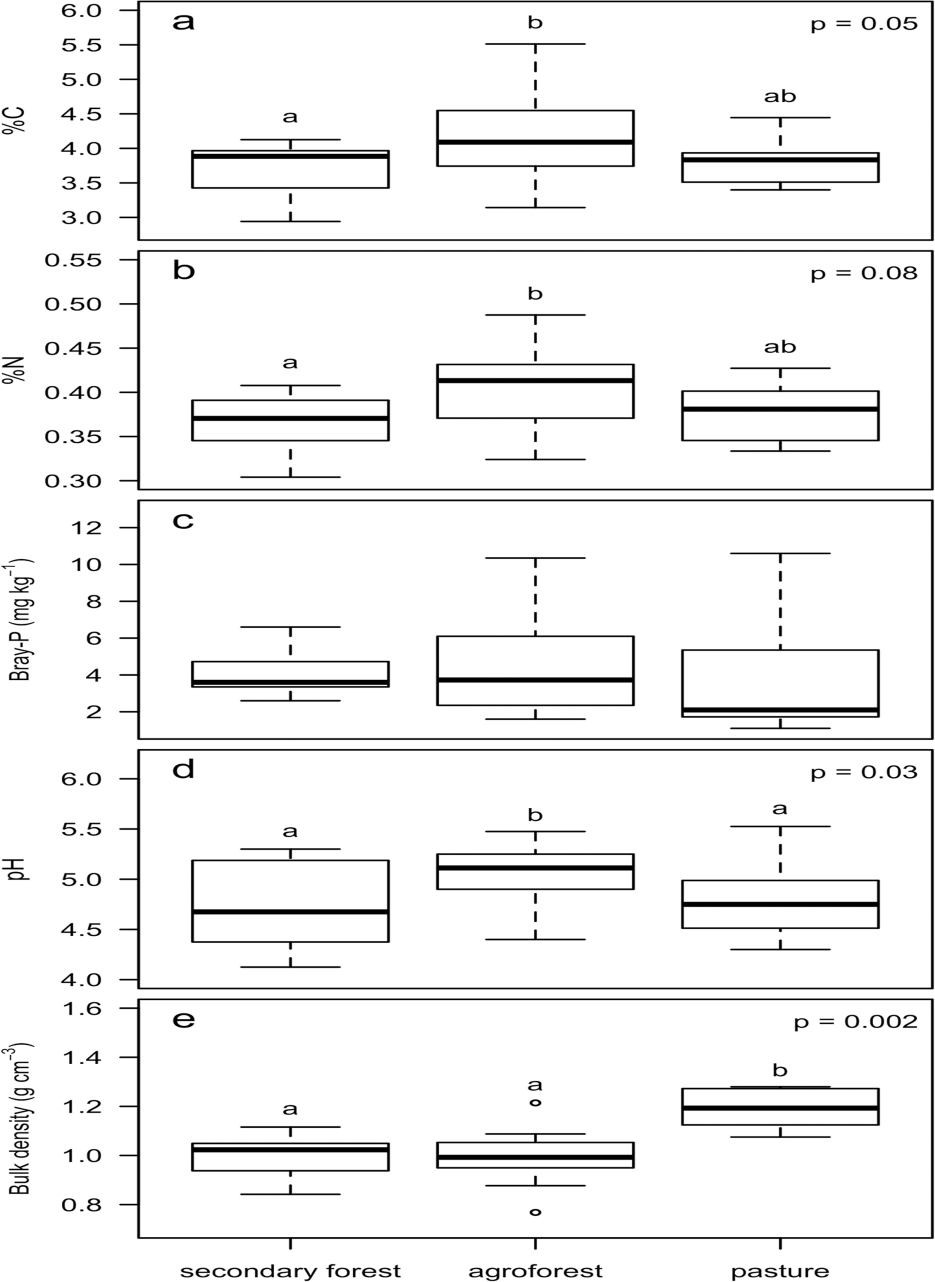
Relationship between land use type and surface soil qualities. Relationship between land use type (secondary forest (N = 8), agroforest (N = 17) and pasture (N = 7)) on soil %C (a), %N (b), Bray-P (c), pH (d), and bulk density (e). Non-overlapping letters signify significant difference at Non-overlapping letters signify significant difference at α ≤ 0.05 for %C and ≤ 0.1 for %N.

Soil %C had a statistically significant relationship with land use type (p = 0.05) and soil %N was marginally affected by land use type (p = 0.08), whereas there was no significant relationship was detected between land use and available P (Fig 8). Tukey HSD post hoc comparisons indicated that agroforest had significantly greater %C and %N than secondary forest in the top 10 cm of the soil, but pasture did not significantly differ from either agroforest or secondary forest (Fig 8A–8C). Land use also was correlated with soil pH (p = 0.03); Tukey HSD post hoc comparisons indicated that agroforest soil was less acidic than both secondary forest and pasture, which did not significantly differ from each other (Fig 8D). Soil bulk density was correlated with land use (p = 0.002). Tukey HSD post hoc comparisons indicated that pasture had a significantly greater bulk density than either secondary forest or agroforest, which did not differ from each other (Fig 8E). When data for soil nutrients and morphospecies richness from sites in all land-use types were pooled, we observed no effect of morphospecies richness or phylodiversity on any soil property.

## Discussion

We found that agroforestry sites may be comparable to unmanaged secondary forest across a variety of ecosystem functions and properties. Because tropical agricultural conversion causes considerable primary habitat and species loss [58], conservation efforts generally focus on closed-habitat species and their frontier forest ecosystems [59]. However, our results suggest that well-established agroforests composed of perennial tree and shrub crops intermixed with non-crop trees have the potential to be comparable in both species richness and phylodiversity to uncultivated secondary forests (Fig 4A and 4B). Managed agroforests were sometimes more speciose at the plot scale than secondary forests, but these species largely differed among land use types. While comparable to some studies [60], our pasture systems had a lower average species diversity than has been observed in other neotropical sites [45], which may reflect differences in pasture maintenance and landscape heterogeneity. Notably, pasture species richness and phylodiversity were significantly lower than secondary forest and agroforest, while the NRI of agroforest and pasture was greater than secondary forest (but did not differ from each other, Fig 4C). This pattern suggests that despite evidence that agroforests may support a similar species richness and phylodiversity to unmanaged secondary forests, active human management of agroforests appears to select for more closely related species.

Despite minimal active management in secondary forests, potential natural resource diversity tended to be comparable between the secondary forest and agroforest, while it was lowest in the pasture (Fig 5). Agroforests had an even mixture of single-, double-, and triple-function species, as compared to the secondary forest, which primarily had one- and two-function species (S1 Fig). The relatively high number of species with such high functional utility in actively managed agroforest explains why this system’s overall functional value was comparable to secondary forests despite having slightly lower species richness and phylodiversity, both of which positively correlate with functional diversity (Fig 7). Nonetheless, unmanaged secondary forests remain critical, as they are home to the species predominantly used for lumber, in addition to having comparable potential functional value of both food and medicine as agroforests (Fig 6). However, our results point to the utility of maintaining a matrix of accessible secondary forests alongside agroforests to promote both plant biodiversity conservation and socioeconomic development.

Complementing our assessment of aboveground natural resource diversity, we documented the influence of land use type on soil factors which are critical for regulating the productive potential and ecological functions of agroforestry systems [61]. The traditional practice of adding husks and other plant residues back to agroforestry sites–in addition to slash and burn land clearing, which transiently raises soil pH [61]–may increase soil pH while also enriching soil nutrient status [62]. Although the top 10 cm of soil in the well-developed agroforestry sites surveyed had a higher pH than secondary forest or pasture, no influence of land use on plant-available P was detected. However, agroforest soil was more C- and N-enriched than neighboring secondary forest, and comparable to pastureland (Fig 8). Paralleling past studies, pasture also had a higher bulk density relative to agroforest (and secondary forest), which can reduce soil aeration, porosity, and permeability, thereby negatively affecting fertility [60]. Therefore, established agroforestry systems tend to maintain soil structure relative to pastureland, which can increase nutrient cycling efficiency, and minimize leaching losses, a primary cause of soil fertility declines in annual cropping and pasture systems [61]. No significant relationships were identified between plant diversity and soil properties when pooled across land use types suggesting that land use decisions (such as adding back plant residues) may influence soil properties more strongly than plant species diversity.

Soil P limitation is a common phenomenon in old Neotropical forest soils; low available P-availability is a commonly noted constraint in tropical agriculture [63]. All land use types assessed had available P values significantly below proposed critical levels (the available P concentrations correlated with 80% maximum crop yield in tropical Oxisols, Ultisols, and Alfisols) of 7–10 mg kg^-1^ [64]. Similar to other studies in P-poor tropical agroforests [61], land use choice appears to have little influence on available soil P in this lowland tropical system. The low available P concentrations observed across soil types suggest that P limits plant growth and agricultural yield in both unmanaged and managed land use types. Recommendations for stimulating productivity should therefore include the addition of P-rich organic matter.

Agroforestry systems, which are within the scope of the Kyoto Protocol sanctioned Clean Development Mechanism intended to increase C sequestration while improving land quality in deforested or degraded lands, are well-documented to have higher potential to sequester aboveground C than pastures and field crops [18,21,65–67]. Less emphasis, however, is typically placed on other functions that agroforestry systems may support. Our study suggests that agroforestry systems have the potential to be comparable to unmanaged secondary forests in their biological and natural resource diversity. This finding does not imply, however, that secondary forests are unimportant; secondary forests are home to many species not found in managed systems and can be useful for other socioeconomic functions that may not require direct management strategies.

Allowing agroforestry systems to develop alongside secondary forests and reserve areas may be a viable strategy to promote biodiversity conservation and socio-ecological stability in Atlantic Nicaragua and elsewhere [23,38]. Our results support the paradigm that highly impacted systems such as pasture may be too altered to meaningfully serve socio-ecological functions outside the designed purpose of grazing land [14]. Thus, the expansion of pasture has a much stronger impact on biodiversity and ecosystem functions than other management strategies while conferring few other socio-ecological benefits. By coupling a suite of ecological and social metrics, we were able to more comprehensively assess the impacts of common land use strategies in a rapidly developing area of Atlantic Nicaragua on terrestrial ecosystems. An integrative approach like the one employed here may strengthen future assessments of the tradeoffs associated with different land use strategies in this region and elsewhere.

## Supporting Information

S1 Fig Proportion of species that have 1 or more functions in each land use studied. Proportion of species that have 1 (a), 2 (b), or 3(c) functions in each land use type. Non-overlapping letters signify significant difference at ∝ ≤ 0.05.
(DOCX)

S1 Table Sample location and raw soil and plant data. (XLSX)

S2 Table Plant species functions. (XLSX)

S3 Table Plant species or morphotypes found in each land use type. (XLSX)
