# Supplementary figures and images for: Agroforestry Practices Promote Biodiversity and Natural Resource Diversity in Atlantic Nicaragua
Source: PLoS One. 2016 Sep 8;11(9):e0162529. doi: 10.1371/journal.pone.0162529 (PMC5015841; doi:10.1371/journal.pone.0162529)

Figure 1


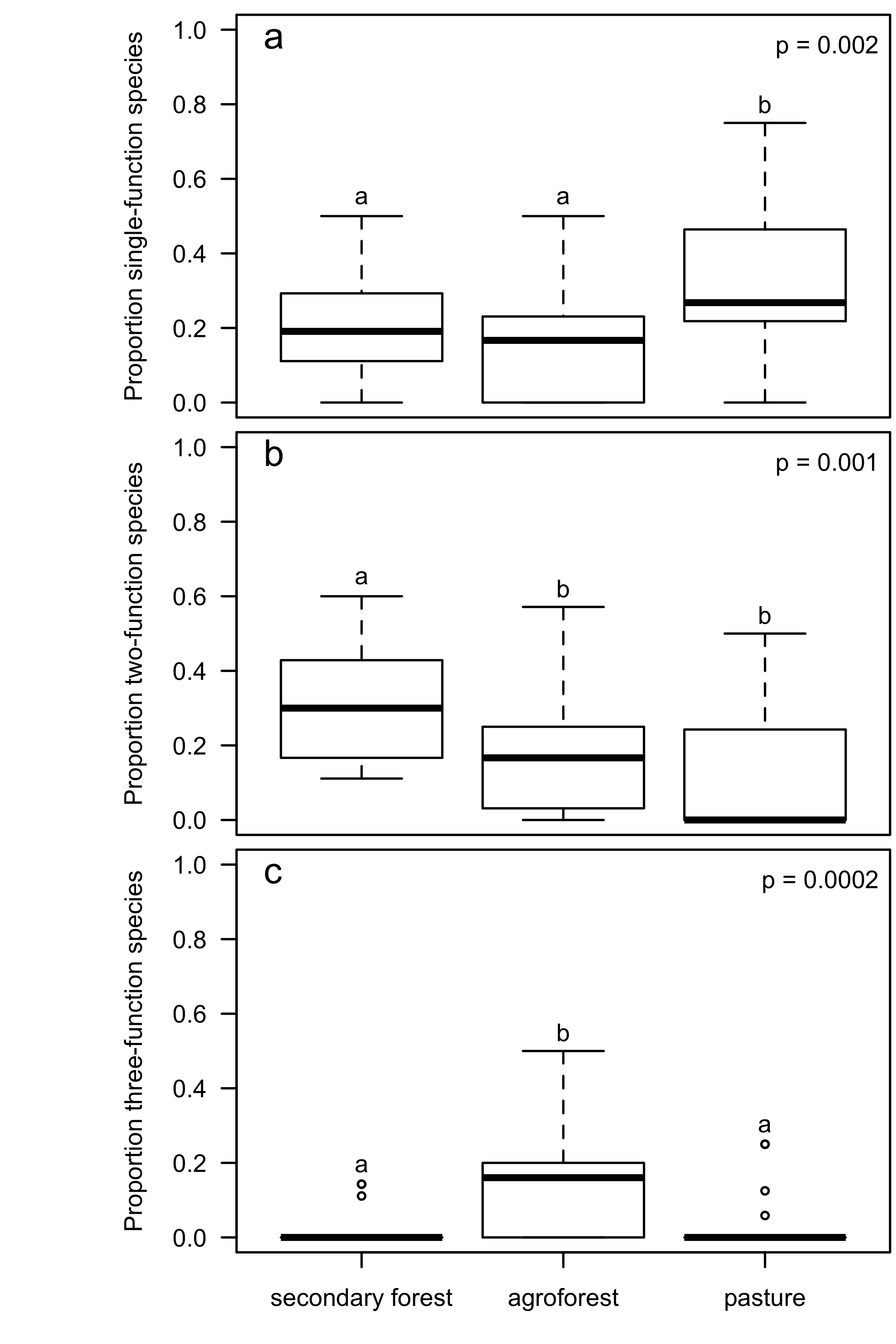

Supplement: S1 Fig — Proportion of species that have 1 (a), 2 (b), or 3(c) functions in each land use type. Non-overlapping letters signify significant difference at ∝ ≤ 0.05. (DOCX) [file pone.0162529.s001.docx]
